# Assessing diversity of the female urine microbiota by high throughput sequencing of 16S rDNA amplicons

**DOI:** 10.1186/1471-2180-11-244

**Published:** 2011-11-02

**Authors:** Huma Siddiqui, Alexander J Nederbragt, Karin Lagesen, Stig L Jeansson, Kjetill S Jakobsen

**Affiliations:** 1 University of Oslo, Department of Biology, Centre for Ecological and Evolutionary Synthesis, P.O. Box 1066 Blindern, 0316 Oslo, Norway; 2 University of Oslo, University Hospital HF Ullevaal-Oslo and Faculty of Medicine, Department of Microbiology, P.O. Box 4956 Nydalen, 0424 Oslo, Norway

## Abstract

**Background:**

Urine within the urinary tract is commonly regarded as "sterile" in cultivation terms. Here, we present a comprehensive in-depth study of bacterial 16S rDNA sequences associated with urine from healthy females by means of culture-independent high-throughput sequencing techniques.

**Results:**

Sequencing of the V1V2 and V6 regions of the 16S ribosomal RNA gene using the 454 GS FLX system was performed to characterize the possible bacterial composition in 8 culture-negative (<100,000 CFU/ml) healthy female urine specimens. Sequences were compared to 16S rRNA databases and showed significant diversity, with the predominant genera detected being *Lactobacillus*, *Prevotella *and *Gardnerella*. The bacterial profiles in the female urine samples studied were complex; considerable variation between individuals was observed and a common microbial signature was not evident. Notably, a significant amount of sequences belonging to bacteria with a known pathogenic potential was observed. The number of operational taxonomic units (OTUs) for individual samples varied substantially and was in the range of 20 - 500.

**Conclusions:**

Normal female urine displays a noticeable and variable bacterial 16S rDNA sequence richness, which includes fastidious and anaerobic bacteria previously shown to be associated with female urogenital pathology.

## Background

Microbes, including bacteria, viruses and protists, reside both on the surface and deep within numerous sites in the human body. It is estimated that trillions of microorganisms inhabit the average healthy human and that microbial cell counts in and on the human body outnumber the human cells by a factor of 10 [1,2]. Studies confirm that humans live in a symbiosis with most of these microbes, whose roles span from harmless to important to life and health [1,3,4]. However, microorganisms can also be detrimental to their host and cause diseases such as digestive disorders, obesity, skin diseases, oral disease, bacterial vaginosis (BV), sexual transmitted diseases and urinary tract infections (UTI) [2,5-9].

Urine within the urinary tract has in general been considered sterile [10,11], based upon a lack of culturable microbial cells present in urine specimens obtained by the clean-catch method and by catheterization [12-15]. Confirmation of a UTI relies on demonstrating significant bacteriuria (or funguria) in a voided midstream urine sample. Traditionally, 10^5 ^colony-forming units per ml (CFU/ml) is the threshold for defining a positive (significant) culture result [16,17]. Conventional culturing techniques favor the fast growing and modest bacteria, whereas fastidious bacteria can evade the standard culture conditions [18]. The presence of intracellular bacteria in uroepithelial cells [19], and even biofilm formation in the urinary tract has been suggested [20,21]. Investigation of healthy urine specimens has demonstrated the presence of non-culturable bacterial cells [22]. These findings stress that bacteria present in urine specimens can escape detection by culture-dependent methods, and that the current view of bacterial diversity in urine thus may be incomplete. This leaves a cryptic fraction of bacteria that may be explored by other means.

Culture-independent, 16S ribosomal DNA (rDNA) sequencing has been widely utilized in the past two decades to study bacterial diversity from various habitats since sequencing of PCR-amplified 16S rDNA overcomes the limitations of culture-based bacterial detection [23]. However, often the search for microbial agents is performed only after a disease state has been diagnosed. Only a few investigations including urine from healthy persons using 16S rDNA PCR have been reported [12,24-26]. These studies had a variable success rate in actually obtaining sequences, resulting in a limited overview of the healthy urine bacterial flora. However, two recent 16S rDNA studies by Nelson *et al*. (2010) and Dong *et al*. (2011) [27,28] have shown that the male urine contains multiple bacterial genera.

Advances in sequencing technology, such as massively parallel pyrosequencing as developed by 454 Life Sciences [29], allow for extensive characterization of microbial populations in a high throughput and cost effective manner [30,31]. Amplicons of partial 16S rRNA genes are sequenced on microscopic beads placed separately in picoliter-sized wells, bypassing previously needed cloning and cultivation procedures. Such sequencing has revealed an unexpectedly high diversity within various human-associated microbial communities, e.g. oral-, vaginal-, intestinal- and male first catch urine microbiota [4,28,32,33], but female urine microbial diversity has so far not been studied using high throughput sequencing (HTS) methods.

Here, we have investigated the bacterial diversity in urine microbiota from healthy females by means of 16S rDNA amplicon 454 pyrosequencing. This study demonstrates the use of this methodology for investigating bacterial sequence diversity in female urine samples. Our results indicate a diverse spectrum of bacterial profiles associated with healthy, culture negative female urine and provide a resource for further studies in the field of molecular diagnostics of urine specimens.

## Methods

### Urine sampling

Urine was collected by the clean catch method in which healthy adult female volunteers (n = 8), collected midstream urine into a sterile container. Specimens were initially kept at 4°C, and within an hour transported to the laboratory for DNA isolation. All specimens were culture negative, as tested by the Urological Clinic at the University Hospital HF Aker-Oslo. Samples were taken with informed consent and the study was approved by the Regional Committee for Medical Research Ethics East-Norway (REK Øst Prosjekt 110-08141c 1.2008.367).

### DNA isolation

30 ml urine volume was pelleted by centrifugation at 14000 RCF for 10 min at 4°C. 25 ml of the supernatant was decanted and the pellet was resuspended in the remaining volume. 5 ml of the sample was again pelleted by centrifugation for 10 min at 16000 × g (4°C). The pellet and some supernatant (up to 100 μl) were processed further. DNA was isolated from the urine pellets with DNeasy Blood & Tissue kit (QIAGEN, Germany), following the tissue spin-column protocol with minor modifications. Briefly, cell lysis was initiated by adding 100 μl POWERlyse lysis buffer (NorDiag ASA, Oslo, Norway) followed by incubation at 80°C for 10 min. Finally, 200 μl of Qiagen buffer AL was added. Samples were mixed by pulse-vortexing for 15 sec. From this point onward, purification was carried out as per manufacturer's instructions. Finally, the DNA was eluted in 100 μl of AE buffer from the kit. The DNA concentrations in the samples were measured by using the Quant-iT PicoGreen dsDNA assay kit (Molecular Probes, Invitrogen USA) and ranged from 0.33 ng/μl to 1.59 ng/μl.

### 16S rDNA PCR

DNA (10 μl of 1:9 dilution) was amplified by PCR using the broad range 16S rDNA primers described in Table 1. The composite primers each comprised a 17-20 bases target specific region at their 3' end and a 19 bases region of the Primer A (forward primer) or the Primer B (reverse primer) sequences needed for GS FLX amplicon sequencing (454 Life Sciences, USA) at their 5'end. PCR reactions were performed using 25 μl (final volume) mixtures containing 1× GeneAmp PCR Gold Buffer Applied Biosystems, 3.5 mM MgCl_2_, 0.2 mM GeneAmp dNTP, 10 pmol of each primer and 0.025 U/μl AmpliTaq Gold DNA Polymerase, LD (Applied Biosystems, USA). The amplification protocol for the V1V2 amplicon primers was: 95°C for 10 min, followed by 35 cycles of 95°C for 30 s, 50°C for 30 s and 72°C for 25 s, and a final elongation step at 72°C for 7 min. The protocol for the V6 amplicon primers was: 95°C for 10 min, followed by 35 cycles of 95°C for 30 s, 50°C for 25 s and 72°C for 25 s, and a final elongation step at 72°C for 7 min. Replicate PCRs were performed for each sample. A positive control (with previously amplified bacterial DNA) as template was run for every PCR.

**Table 1:** PCR primers used

Primer	Sequence (5'→3')	16S rDNA region	Product size	Reference
A^2^+**V1 F**	GCCTCCCTCGCGCCATCAG**AGAGTTTGATCMTGGCTCAG**	V1V2	392 bp**^3^**	[32]
B^2^+**V2 R**	GCCTTGCCAGCCCGCTCAG**CYNACTGCTGCCTCCCGTAG**	8-361**^1^**		
				
A^2^+**1061R**	GCCTCCCTCGCGCCATCAG**CRRCACGAGCTGACGAC**	V6	316 bp**^3^**	[33]
B^2 ^+**784F**	GCCTTGCCAGCCCGCTCAG**AGGATTAGATACCCTGGTA**	784-1061**^1^**		

The table contains primer name, sequence (hypervariable specific sequence in bold font), 16S rDNA region covered, product size and references for the primers used in this study.

**^1 ^**Coordinates are given relative to the 1542 bp *E. coli *K12 16S rDNA sequence.

**^2^**A and B primer: corresponds to 454-adaptor sequences from the amplicon pyrosequencing protocol for GS FLX http://www.my454.com/downloads/protocols/Guide_To_Amplicon_Sequencing.pdf[101], p. 7.

**^3^**Product size includes the primer sequences.

PCR amplicons were detected and confirmed for DNA from all eight subjects by agarose gel electrophoresis prior to pyrosequencing (data not shown).

All crucial steps during DNA isolation and the entire PCR set up were performed in a laminar air flow (LAF)-bench, illuminated with a UV lamp prior to use in order to avoid possible contaminants. In addition, negative DNA extraction controls (lysis buffer and kit reagents only) were amplified and sequenced as contamination controls.

Additionally, negative PCR controls (sterile Molecular Biology Grade Water from 5PRIME (VWR, Norway) as template) were run for every PCR protocol, resulting in no PCR product.

### 454 pyrosequencing

Replicate PCR products were pooled and purified using Agencourt AMPure PCR purification (Beckman Coulter, USA). DNA concentration and quality were assessed on a Bioanalyzer 2100 (Agilent, USA). Equal amounts of both amplicons (V1V2 and V6) for a single subject or contamination control were pooled and sequenced using GS FLX chemistry in the same lane of a PicoTiterPlate divided into 16 lanes. Each of the amplicons was pyrosequenced together, except for samples F1 and F3.

454 pyrosequencing was performed by the Norwegian Sequencing Centre (NSC) at the Department of Biology, University of Oslo, Norway.

### Sequence read analysis

A total of 190 287 reads were produced (female urine 165 041 raw reads and contamination control 25 246 raw reads). The initial sequence reads were split into two pools using the V1V2 and V6 primer sequences via the sfffile program from 454 Life Sciences, thus reducing the sequences to 152 413 urine reads (Table 2) due to the program splitting on exact match to primer.

**Table 2:** Sampling depth and biodiversity found by amplicon 454 pyrosequencing V1V2 and V6 regions from eight culture negative female urine samples

	*Sample*																	
	*Combined sequence pool*		F1		F2		F3		F4		F5		F6		F7		F8	
	V1V2	V6	V1V2	V6	V1V2	V6	V1V2	V6	V1V2	V6	V1V2	V6	V1V2	V6	V1V2	V6	V1V2	V6
***Sampling depth***																		
Total reads	78346	74067	14579	18362	12629	6565	4305	17474	9877	5005	12645	6586	8216	5692	7861	6986	8234	7397
Length cutoff^1 ^	48861	45382	8479	8039	8416	4752	2721	13066	6253	3467	10116	5074	4428	3047	3967	3495	4481	4442
Denoised ^2^	48860	45136	8479	7977	8416	4703	2721	13064	6253	3461	10116	5057	4427	3031	3967	3432	4481	4411
Cleaned ^3^	48452	44760	8476	7969	8353	4682	2720	13060	6242	3459	10109	5053	4361	2988	3711	3138	4480	4411
Unique OTUs	1354	2069	61	376	456	328	22	115	116	102	95	81	523	134	322	581	163	538
OTUs^4 ^3%	1209	1435	52	240	411	254	20	81	101	85	73	63	504	116	300	499	130	338
OTUs^4 ^6%	1092	1072	50	178	379	210	19	61	92	73	62	51	472	101	270	436	116	244
Phyla^5 ^(11)	10	8	4	4	6	3	1	3	4	4	3	3	3	4	8	7	4	4
Genera^5 ^(45)	35	28	8	8	15	10	1	8	10	5	6	4	4	4	19	17	9	8
																		
***Diversity indices***																		
Chao1^6 ^(3%)	1211	2469	64.75	456.36	412.62	410.33	24.5	128.83	104	195.5	86.04	108.76	504.11	130.6	324.6	1121.43	250.12	835.02
Chao1 LCI95	1209	2286	56.13	371.05	411.36	353.85	20.97	102.95	101.7	136.49	77.88	82.43	504	122.1	313.14	953.17	195.84	670.9
Caho1 HCI95	1216	2690	91.27	597.21	418.2	498.76	40.69	185.2	112.75	322.11	107.8	170.8	506.28	148.39	346.03	1352.03	349.14	1080.04
Shannon index^7 ^(3%)	2.99	3.05	0.52	1.96	1.99	1.62	0.23	0.49	1.44	1.44	0.33	0.44	3.01	1.32	3.76	4.07	2.06	3.31
Normalized Shannon index (3%) ^8^			0.52	1.96	1.86	1.63	0.23	0.50	1.42	1.44	0.34	0.45	2.89	1.35	3.72	4.07	2.06	3.31

^1^Length cutoff at minimum 218 nt for V1V2 reads and 235 nt for V6 reads.

^2^Total number of sequences after processing the dataset through the PyroNoise program developed by Quince *et al*., 2009 [34].

^3^The number of reads per dataset after removal of sequences that could be from the same source as those in the contamination control dataset.

^4^OTUs: Operational Taxonomic Units at 3% or 6% nucleotide difference.

^5^Number of phyla and genera are based on taxonomic classification by MEGAN V3.4 [36,37], with the total number of phyla and genera detected in parenthesis.

^6^Chao1 is an estimator of the minimum richness and is based on the number of rare OTUs (singletons and doublets) within a sample.

^7^The Shannon index combines estimates of richness (total number of OTUs) and evenness (relative abundance).

^8^The Shannon index after normalization of the number of sequences (as described in Methods).

The 454 pyrosequencing method has a characteristic error rate in the form of insertion/deletion errors at homopolymer runs. To correct for this phenomenon, the raw reads were processed with PyroNoise [34] with a minimum length cutoff of 218 and 235 nt for the V1V2 and V6 regions, respectively. The PyroNoise program clusters all reads whose flowgrams indicate that they could stem from the same sequence, while also considering read abundance. After denoising, one sequence per cluster together with the number of reads mapping to that cluster is reported. Next, the sequences (at this stage one sequence per denoised cluster) that did not have an exact match to the primer were removed, and the forward primer sequence itself was also trimmed. Finally, the urine sample sequence sets were stripped for sequences that could be from the same source as those in the contamination control dataset. This was done by using the program ESPRIT http://www.biotech.ufl.edu/people/sun/esprit.html[35] to do a complete linkage clustering at 1% genetic difference of each sample together with its respective control. Before clustering, the control sequences were weighed so that there were the same number of reads stemming from both the sample and the control going into the process. Within each cluster the frequency of sample vs control sequence was calculated, and any sample sequences found in clusters where 50% or more of the sequences belonged to the control were removed.

For taxonomic grouping we used MEGAN V3.4 http://www-ab.informatik.uni-tuebingen.de/software/megan/welcome.html[36,37], which uses blast hits to place reads onto a taxonomy by assigning each read to a taxonomic group at a level in the NCBI taxonomy. The sequence reads (one read per denoised cluster from the pyronoise step) that passed the filtering steps were compared to a curated version of the SSUrdp database [38] using blastn with parameters set to a maximum expectation value (E) of 10^-5^. The 25 best hits were kept. To reflect abundance behind each denoised sequence cluster, prior to taxonomic classification each entry in the blast output file was replicated as many times as there were reads mapping to its query sequence. MEGAN analysis of these blast records was performed using a minimum alignment bit score threshold of 100, and the minimum support filter was set to a threshold of 5 (the minimum number of sequences that must be assigned to a taxon for it to be reported). These parameters were consistently used throughout this analysis. When comparing the individual datasets using MEGAN, the number of reads were normalized to 100 000 for each dataset using the compare tool in MEGAN.

Sequences generated in this study have been submitted to the Sequence Read Archive with the study accession number ERP000957. It can be accessed directly through http://www.ebi.ac.uk/ena/data/view/ERP000957.

### Clustering of reads into OTUs

Numbers of operational taxonomic units (OTUs), rarefaction curves, Chao1 richness estimations and Shannon diversities were calculated using MOTHUR v1.17.0 [39], both on each separate sample and on pooled V1V2 and V6 sequences, after replicating each sequence to reflect the amount of reads mapping to its denoised cluster. Each sequence set was first reduced to unique sequences, before a single linkage preclustering step as described by Huse *et al.*, 2010 [40] was performed. In this step, shorter and less abundant sequences were merged with longer and more abundant sequences with a maximum of two differing nucleotides. OTUs were calculated using average clustering at 3%, using a pairwise distance matrix. Distances were calculated using Needleman-Wunsch, discounting endgaps while counting internal gaps separately.

Considering that the Shannon index is sensitive to the original number of sequences generated from a given sample [41] we calculated the Shannon index for normalized numbers of sequences for each separate sample. A random number of reads, corresponding to the lowest number of sequences in a sample group, i.e. 2720 for V1V2 and 2988 for V6, were picked 100 times from each sequence set. These new sequence sets were processed through MOTHUR in the same fashion as the full sequence sets and the average of the resulting Shannon values are shown in Table 2.

## Results

### 454 pyrosequencing data

In our study a total of 78 346 sequences for the V1V2 region and 74 067 sequences for the V6 region were obtained (Table 2). The quality filtering approach as described in Methods eliminated 40% of the sequenced reads. Additionally, since the bacterial identification technique (broad range 16S rDNA PCR) utilized in this study was highly sensitive and susceptible to environmental contamination, we included negative control extractions, followed by PCR and sequencing, to determine the contamination resulting from the chemicals and consumables used. The read datasets were stripped for sequences found to cluster predominantly with contamination control sequences. This resulted in removal of an additional 1% of the reads, showing that background contamination levels were low (Table 2).

### Identity of the bacterial DNA found in female human urine

**Figure 1:**
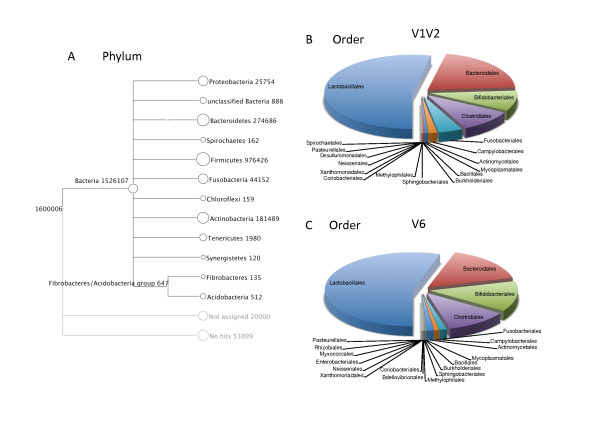
**Summary of the microbial phyla and orders detected in human female urine**. **A: **An overview of the taxonomy at the phylum level as computed using MEGAN V3.4, using normalized counts by pooling together the V1V2 and V6 16S rDNA reads. The size of the circles is scaled logarithmically to the number of reads assigned to the taxon. Nodes denoted as "Not assigned" and "No hits" are the number of reads that were assigned to a taxon with fewer than 5 hits, or did not match to any sequence when compared to the SSUrdp database, respectively. **B and C: **Comparison of taxonomic assignments for human female urine sequences at the order level. Reads obtained using the V1V2 hypervariable 16S rDNA region were predominantly assigned to *Lacobacillales*, and identified in total 18 different orders where *Desulfuromonadales *and *Spirochaetales *are unique to this V1V2 dataset. V6 reads revealed a slightly higher diversity with 20 different orders; *Bdellovibrionales, Myxococcales, Rhizobiales *and *Enterobacteriales *are only identified by this V6 method.

An analysis using MEGAN of all pooled reads from the two different amplicon libraries of the 16S rRNA gene (i.e. V1V2 and V6 regions) revealed a total of eleven phyla in female urine, with the bacterial DNA sequences predominantly found in *Firmicutes *(65%), *Bacteroidetes *(18%), *Actinobacteria *(12%), *Fusobacteria *(3%), and *Proteobacteria *(2%) (Figure 1A). The other 6 phyla were represented by less than 1% of the total sequence reads. The phylum *Chloroflexi *was identified by only the V6 sequence dataset; similarly, the phyla *Spirochaetes, Synergistetes *and *Fibrobacteres *were only identified by the V1V2 sequence dataset.

When examining the two sequence sets separately, 22 different orders were identified in total. The 4 most abundant bacterial orders were the same for both regions sequenced; *Lactobacillales *(53% for V1V2 and 55% for V6), *Bacteroidales *(20% for V1V2 and 16% for V6), *Clostridiales *(10% for V1V2 and 11% for V6), and *Bifidobacteriales *(9% for V1V2 and 13% for V6) (Figure 1B and 1C). Additionally, 18 other orders were detected in both the V1V2 and V6 datasets. Further, *Bdellovibrionales, Myxococcales*, *Rhizobiales *and *Enterobacteriales *were only identified in the V6 sequence dataset, while *Desulfuromonadales *and *Spirochaetales *were only observed in the V1V2 dataset (Figure 1B and 1C).

**Figure 2:**
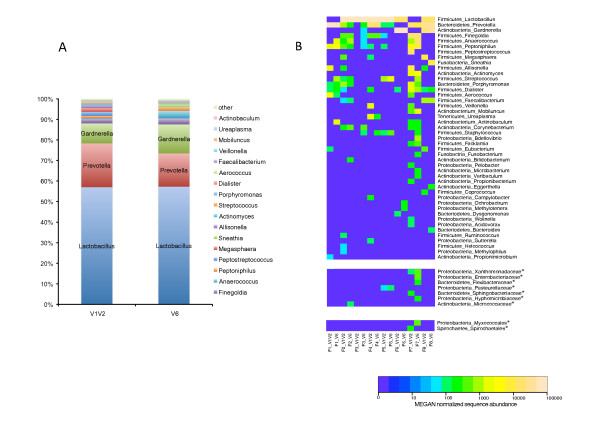
**Bacterial genera detected in healthy female urine**. **A: **Comparison of healthy female urine bacterial genera abundance determined by sequencing 2 different hypervariable 16S rDNA regions, V1V2 and V6. Relative abundance of 18 major bacterial genera found in the sequence pool of eight different urine samples are shown for the two 16S rDNA regions. Groups denoted "other" represent minor groups classified. Y-axis represents relative abundance. **B**: Heat map showing the relative abundance of bacterial genera across urine samples of eight healthy females. Genera denoted as phylum_genus, samples denoted as samplenumber_V1V2 or V6. Taxa marked with asterisk (*) could not be assigned to any genera, and are shown at the lowest common taxon: family and order. Color intensity of the heat map is directly proportional to log 10 scale of the abundance normalized sequence data as done by MEGAN.

Analyzing the data at the genus level revealed 45 different genera. 88% and 87% of the reads in the V1V2 and V6 sequence datasets, respectively, were assigned to *Lactobacillus, Prevotella *and *Gardnerella *(Figure 2A). These three major genera found in female human urine belong to the three most predominantly detected phyla: *Firmicutes, Bacteroidetes *and *Actinobacteria *(Figure 1A). Out of the 45 different genera, 17 genera were unique for the V1V2 sequence reads, whereas a total of 10 genera were uniquely found with V6 sequence reads.

Keeping the same parameters as for the analysis at higher taxonomic levels, a small number of bacterial reads from the V1V2 and V6 dataset were assigned to species level, see Additional file 1: Table S1. When comparing to previous reports from literature [9,17,37,42-81], nine out of the 45 species listed are associated with UTI. Twenty of the species listed represent uncultured bacteria, many of them with an unknown pathogenic potential (Additional file 1: Table S1).

### Variation between urine samples from different individuals

The distribution of the different taxa differed markedly among the urine specimens. 16S rDNA sequences from the phyla *Firmicutes *and *Bacteroidetes *were found in all urine samples. Sequences from *Proteobacteria *and *Actinobacteria *were observed in 6/8 and 5/8 urine samples respectively, while sequences from *Fusobacteria *were identified in only 2 samples. The remaining six phyla defined in our pooled urine sequence dataset were only detected once among the urine samples; *Spirochaetes, Chloroflexi, Fibrobacteres *and *Acidobacteria *in sample F7, *Tenericutes *in sample F4 and *Synergistetes *in sample F2. These results indicate that there is a noticeable intra-individual variation in urine 16S rDNA sequences even at the phylum level.

The interpersonal microbial sequence diversity and the distribution of bacterial DNA at the genus level in each individual are shown in the heat map in Figure 2B. In the majority of the urine specimens (6 out of 8) one genus was dominant, i.e. represented by at least 75% of the reads, while in two specimens (sample F7 and F8) there was a more even distribution among the represented genera (Figure 2B). A polymicrobial state is suggested for all but a single urine specimen based on both of the 16S rDNA sequence datasets. The exception was sample F3, which showed only the presence of *Lactobacillus *based on the V1V2 reads, while the V6 amplicon sequence data identified seven additional bacterial genera, though at a low frequency. The most frequently identified genus was *Prevotella*, with sequences present in 7 out of 8 urine samples. Sequences assigned to *Lactobacillus*, *Peptoniphilus *and *Dialister *were also frequently detected (6/8), followed by *Finegoldia *(5/8), *Anaerococcus*, *Allisonella*, *Streptococcus*, *Staphylococcus *(all 4/8). Interestingly, reads assigned to *Gardnerella *were only identified in 3/8 urine samples, even though this genus was the 3^rd ^most abundant group in the pooled sequence dataset for both the V1V2 and V6 regions (Figure 2A). Three other genera and a group of 5 genera were identified by reads belonging to 3 or 2 urine samples, respectively. 24 genera were only detected in 1 out of the 8 samples.

### Species richness and diversity estimates of the female urine microbiota

**Figure 3:**
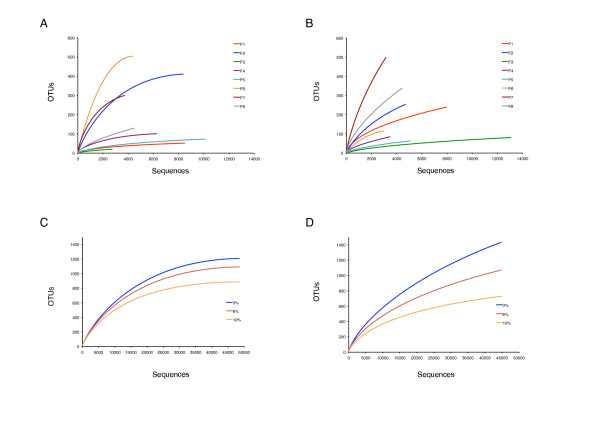
**Number of OTUs as function of the total number of sequences**. **A and B**: Rarefaction curves of individual samples for the V1V2 (A) and the V6 datasets (B). Curves were generated at 3% genetic difference using MOTHUR v1.17.0 [39]. **C and D**: Rarefaction curves of the pooled dataset for both V1V2 reads (C) and V6 reads (D). OTUs with ≤3%, ≤6% and ≤10% pairwise sequence difference generated using MOTHUR v1.17.0 [39] are assumed to belong to the same species, genus and family, respectively.

Bacterial taxonomic richness and diversity varied greatly among urine samples investigated in this study. Community richness and diversity were determined using rarefaction plots, Chao1 and Shannon index estimations (Figure 3 and Table 2).

Rarefaction curves were generated for 3% genetic difference level (e.g., at the species level). The number of OTUs calculated for the eight individual samples ranged from 20-504 and 63-499 OTUs for the V1V2 and V6 regions, respectively (Figure 3A, B and Table 2). OTU numbers of the total bacterial community in the female urine at 3% difference for the V1V2 sequence pool was calculated to 1209 OTUs and to 1435 OTUs for the V6 sequence pool (Figure 3C, D and Table 2). Furthermore, total unique OTUs for the V1V2 pooled reads were 1354 and for the V6 pooled reads 2069 (Table 2).

To compare the diversity between the eight different urine samples, the Shannon diversity index was determined both with the original, and with normalized numbers of sequences (Table 2). There was no substantial difference between the two Shannon indices calculated for the same sample.

## Discussion

In this work we sequenced two different variable regions of 16S rDNA isolated from eight culture-negative urine samples. Urine samples are at risk of contamination by the bacterial flora of the female urogenital system [82,83], therefore sampling of mid-stream urine was performed by the clean catch method, under guidance of an experienced urotherapy nurse. To avoid further bacterial growth, which could skew the results, the samples were kept on ice and analyzed within an hour. Amplicon lengths used here exceed the typical fragment size (150-200 bp) of circulating cell-free DNA in urine [84], thus reducing the frequency of such DNA in our analyses.

### Bacterial profile of female urine

The sequences found in the samples were mainly assigned to the *Firmicutes *phylum (65%) with *Bacteriodetes*, *Actinobacteria*, *Fusobacteria *and *Proteobacteria *members accounting for most of the remaining sequences (Figure 1A). This overall composition of phyla is comparable to prior 16S rDNA sequencing studies of the human urogenital tract (vaginal microbiota [79] and male urogenital tract [27,28,85]). However, we also found sequences from *Fibrobacteres*, a phylum not previously associated with human microbiota as described by the Human Microbiome Project catalog (HMP) [69,86], the Human Oral Microbiome Database (HOMD) [70,87] and in studies on the gastrointestinal tract, vaginal and male urine bacterial flora [27,28,79,88,89].

Our analysis revealed that the bacterial composition in human female urine specimens is polymicrobial and that there is considerable variation between urine samples (Figure 2B). *Lactobacillus, Prevotella *and *Gardnerella *were the dominant genera (Figure 2A), however, not every urine sample exhibited 16S rDNA from these genera (Figure 2B), indicating that a single characteristic microbial community for female urine cannot be established. Similar results were also seen in Nelson *et al*. (2010) [27] and Dong *et al*. (2011) [28] in their studies on male urine composition. While *Lactobacillus *and *Prevotella *were not among the dominant genera in the first study [27], rDNA sequences belonging to these genera were dominant in the latter study [28], as it is in our data. *Lactobacillus *was, however, considerably more abundant in female than in male urine. The two studies on male urine did not display the genus *Gardnerella *(typically associated with the female vagina), as a major bacterium, while this genus is one of three dominating genera in our study. In contrast, *Sneathia*, another vaginal bacterium - only present at low abundance in female urine, was reported as a dominant genus in male urine.

### Comparison of V1V2 and V6 primer sets

Two different primer sets previously used for investigating human microbial communities [32,33] covering different parts of the hypervariable regions were used in this study. The V1V2 region is noted for its robustness for taxonomic classification, while the V6 region is more appropriate for measuring microbial diversity due to high variability [32,90,91]. These differences were also reflected in our study where V1V2 uncovered a wider taxonomical range (Figure 2 and Table 2). Both rDNA regions detected approximately the same groups at phylum and order level, however, a larger difference was evident at the genus level. The V1V2 method detected 35 different genera in total, 16 of which were not found in the V6 dataset. The V6 method detected 28 genera in total, where 10 genera were unique to this dataset. Thus, using a combination of these two primer sets clearly maximized the bacterial diversity that could be detected.

### Estimated species richness in female urine microbiota

Our OTU calculations on female urine displayed richness levels that were in the same range as reported for commensal vaginal microbiota (1584 OTUs) [79], but lower than those reported for oral (3011 to 5669 OTUs) [4,92] and fecal samples (up to 5200 OTUs) [90]. For all but one sample, the Chao1 minimum richness estimates for the V1V2 dataset are in close agreement with the observed number of OTUs (Table 2). In addition, the rarefaction curves approached saturation, demonstrating that the OTU diversity was almost completely covered by the V1V2 variable region (Figure 3A and 3C). In contrast, the Chao1 estimates and the rarefaction curves for all but one of the V6 samples indicated that the current sequencing effort for the V6 variable region was not exhaustive (Table 2 and Figure 3B, D).

### Clinical significance of the bacterial DNA identified in human female urine

The anaerobe microbial profile of urine specimens is not routinely investigated in microbiological laboratories since fastidious bacteria often evade standard culture conditions. The present work shows that, besides bacterial species associated with vaginal, fecal and skin bacterial flora, unsurprising considering the anatomy of the female urogenital tract, several types of bacteria previously not seen in female urine were identified. Interestingly, some species detected have earlier been described as causing UTI and bacterial vaginosis (BV), but here we also detect these potentially pathogenic species in asymptomatic healthy female urine samples. For example, most of the fastidious (opportunistic), mostly anaerobic pathogenic bacteria identified by 16S rDNA PCR and sequencing in a study of UTI samples [9], were also detected in our study. On the other hand, uropathogenic *E.coli *(UPEC), a common cause of UTI [93], was not detected in any of our urine samples.

*Lactobacillus *was dominant in the urine microbiota (see Figure 2A), as it is in the human vaginal microbiota, and all of the other genera previously found in vaginal microbiota were also identified in our samples [64,79]. BV is in a majority of cases characterized by a shift in composition of the vaginal microbial community that results in decreased number of lactic producing bacteria and increased numbers of other facultative or anaerobic species in relation to normal bacterial flora [79]. A similar shift in bacterial composition as seen in BV was found in 4 of our eight urine samples: *Lactobacillus *was either present at a low abundance or not detected at all, and the other genera present were mostly anaerobes. One of these, the anaerobe *Prevotella disiens *is also typically found in females with genital tract infections. Furthermore, the genus *Gardnerella*, comprising only the species *G. vaginalis*, is involved in BV, as well as associated with preterm delivery [94,95], and also reported as an uropathogen [9,96].

Both the species *Aerococcus urinae *and the genus *Ureaplasma*, examples of "difficult-to-culture pathogens" commonly not detectable by conventional culture methods [52], were detected in our samples. *A. urinae *is generally associated with bladder infection in elderly people, but can also cause serious complications, such as infective endocarditis when not detected and treated during UTI diagnosis [97,98]. *Ureaplasma spp *occurs more commonly in patients with symptoms of UTI than previously thought [99], and the species *Ureaplasma urealyticum *has also been associated with chronic urinary symptoms in women [100]. Whether or not these potentially pathogenic bacteria represent non-pathogenetic variants or are simply not causing any disease in this setting remains to be investigated.

## Conclusion

Our finding of sequences of these potentially disease-causing species and genera in healthy female urine is an example of the enhanced resolution that can be obtained by high-throughput sequencing. This study also shows that the urine medium of asymptomatic females is harboring a surprisingly wide range of bacteria, including many potentially associated with pathogenic conditions. Apparently, such bacteria are part of the healthy urine microbiota.

## Authors' contributions

HS, AJN, SLJ and KSJ have contributed to the design of this study; HS processed the samples and carried out laboratory procedures. KL, AJN and HS performed the bioinformatics and taxonomic analyses. HS authored the manuscript and all authors edited and commented on the paper. All authors read and approved the final manuscript.

## Supplementary Material

Additional file 1 **Table S1: Bacteria species identified in female urine by 16S rDNA amplicon 454 pyrosequencing and their general pathogenic potential**. Click here for file
