# Supplementary material for: Assessing diversity of the female urine microbiota by high throughput sequencing of 16S rDNA amplicons
Source: BMC Microbiol. 2011 Nov 2;11:244. doi: 10.1186/1471-2180-11-244 (PMC3228714; doi:10.1186/1471-2180-11-244)
Supplement: Additional file 1 — Table S1: Bacteria species identified in female urine by 16S rDNA amplicon 454 pyrosequencing and their general pathogenic potential. [file 1471-2180-11-244-S1.DOC]

**Table S1: Bacteria species identified in female urine by 16S rDNA amplicon 454 pyrosequencing and their general pathogenic potential**

| ***Taxon^1^*** | ***Species*** | ***Pathogenic potential / localization*** | ***References^2^*** | ***European urinalysis guideline^3^*** | ***HMP^4^*** | ***HOMD^5^*** |
| --- | --- | --- | --- | --- | --- | --- |
| *Actinobaculum* | *Actinobaculum massiliense* | Pathogen, chronic UTI | [44] |  | Skin |  |
|  | *Actinobaculum schaalii* | Pathogen, UTI | [48] |  | Skin |  |
| *Aerococcus* | *Aerococcus christensenii* | Unknown, VF | [42] |  | Urogenital tract |  |
|  | *Aerococcus sanguinicola* | Pathogen, UTI | [46] |  |  |  |
|  | *Aerococcus urinae* | Pathogen, UTI | [52] | Doubtful pathogen | Urogenital tract |  |
| *Anaerococcus* | *Anaerococcus octavius* | None, VF | [43] |  | Skin |  |
|  | *Anaerococcus sp. gpac155* | None, HF | [51] |  |  |  |
|  | *uncultured Anaerococcus sp.* | Unknown, EF | TaxID 293428 |  |  |  |
| Fam*.Campylobacteraceae* | *Bacteroides ureolyticus* | Pathogen, BV, UTI | [9, 45] |  | Skin | X |
| *Campylobacter* | *Campylobacter hominis* | Enteropathogen, FF | [49] |  |  |  |
| *Coprococcus* | *Coprococcus eutactus* | None, FF | [47] |  | Gastrointestinal tract |  |
| *Corynebacterium* | *Corynebacterium pseudogenitalium* | Pathogen, UTI | [50] |  | Urogenital tract |  |
| *Eubacterium* | *Eubacterium sp. oral clone BU061* | Unknown, OF | [53] |  |  |  |
| *Facklamia* | *Facklamia hominis* | Possible, VF | [55, 58] |  | Gastrointestinal tract/Urogential tract/Skin |  |
| *Faecalibacterium* | *Faecalibacterium prausnitzii* | Protective, FF | [57] |  | Gastrointestinal tract |  |
| *Finegoldia* | *Finegoldia magna* | Opportunistic, SF, UTI | [9, 60] |  | Urogenital tract | X |
| Phylum *Synergistetes* | *Flexistipes sp. E3_33* | Endodontic infection, OF | [61] |  |  |  |
| *Lactobacillus* | *Lactobacillus coleohominis* | Nonpathogenic, VF | [63] | Nonpathogenic | Urogenital tract | X |
|  | *Lactobacillus crispatus* | Nonpathogenic, VF | [64] | Nonpathogenic | Urogenital tract |  |
| *Methylotenera* | *Methylotenera mobilis* | Unknown, EF | [59] |  |  |  |
| *Peptoniphilus* | *Peptoniphilus sp. gpac121* | Commensal, HF | [51] |  |  |  |
|  | *uncultured Peptoniphilus sp.* | BV | [62] |  |  |  |
| *Peptostreptococcus* | *uncultured Peptostreptococcus sp.* | Odontogenic infection, OF | [65] |  |  |  |
| *Porphyromonas* | *Porphyromonas somerae* | Chronic skin infection, SF | [68] |  | Skin |  |
|  | *uncultured Porphyromonas sp.* | Odontogenic infection, OF | [65] |  |  |  |
| *Prevotella* | *Prevotella corporis* | OF | [66] |  | Urogenital tract |  |
|  | *Prevotella disiens* | OF /pathogenic female genitourinary tract, UTI | [9, 17, 66] |  | Urogenital tract |  |
|  | *uncultured Prevotella sp.* | Odontogenic infection, OF | [65] |  |  |  |
| *Propionimicrobium* | *Propionimicrobium lymphophilum* | Pathogenic, mucosal surfaces, UTI | [9, 67] |  | Urogenital tract |  |
| Phylum *Acidobacteria* | *uncultured Acidobacteria bacterium* | Unknown, EF | TaxID 171953 |  |  |  |
| *Allisonella* | *uncultured Allisonella sp.* | Normal skin microbiota, SF | [56] |  |  |  |
| *unclassified Bacteria* | *uncultured candidate division OD1 bacterium* | Unknown, EF | TaxID 221218 |  |  |  |
|  | *uncultured candidate division OP11 bacterium* | Unknown, EF | TaxID 174923 |  |  |  |
| Phylum *Chloroflexi* | *uncultured Chloroflexi bacterium* | Unknown, EF | [54] |  |  |  |
| Order *Clostridiales* | *uncultured Clostridiaceae bacterium* | Part of gut ecosystem, FF | [74] |  |  |  |
|  | *uncultured Clostridiales bacterium* | Part of gut ecosystem, FF | [76] |  |  |  |
| Order *Sphingobacteriales* | *uncultured Cytophagales bacterium* | Supragingival biofilm, OF | [75] |  |  |  |
| *Eggerthella* | *uncultured Eggerthella sp.* | BV | [79] |  |  |  |
| Phylum *Fibrobacteres* | *uncultured Fibrobacteres bacterium* | Bacterial soil community, EF | [81] |  |  |  |
| *Megasphaera sp.* | *uncultured Megasphaera sp.* | BV | [79] |  |  |  |
| *Methylophilus* | *uncultured Methylophilus sp.* | Normal skin microbiota, SF | [73] |  |  |  |
| Class *Betaproteobacteria* | *uncultured Neisseriaceae bacterium* | Opportunistic, OF | [78] |  |  |  |
| *Pelobacter* | *uncultured Pelobacter sp.* | EF | [72] |  |  |  |
| *Veillonella* | *uncultured Veillonella sp.* | Normal salivary microbiota, OF | [77] |  |  |  |
| *Wolinella* | *Wolinella succinogenes* | OF | [80] |  |  |  |

Abbreviations: UTI: urinary tract, BV: bacterial vaginosis, FF: faecal flora, SF: skin flora, OF:oral flora, EF: environmental flora, VF: vaginal flora, HF: human flora

^1^Taxa as assigned by MEGAN [37], taxon level is genus unless otherwise specified.

^2^References as listed in the main manuscript.

^3^European urinalysis guidelines [71]

^4^Human Microbiome Project [http://www.hmpdacc.org](http://www.hmpdacc.org/) [69]

^5^Human Oral Microbiome Database [http://www.homd.org](http://www.homd.org/) [70]

X: species also described in HOMD
